# Decreased Expression of T-Cell Costimulatory Molecule CD28 on CD4 and CD8 T Cells of Mexican Patients with Pulmonary Tuberculosis

**DOI:** 10.1155/2010/517547

**Published:** 2010-09-08

**Authors:** German Bernal-Fernandez, Patricia Espinosa-Cueto, Rosario Leyva-Meza, Nathalie Mancilla, Raul Mancilla

**Affiliations:** ^1^Facultad de Farmacia, Universidad Autonoma del Estado de Morelos, 62209 Morelos, Mexico; ^2^Departamento de Inmunologia, Instituto de Investigaciones Biomedicas, Universidad Nacional Autonoma de Mexico, Apartado Postal 70228 Ciudad Universitaria, 04510, Mexico City, Mexico

## Abstract

Patients with tuberculosis frequently develop anergy, a state of T-cell hyporesponsiveness in which defective T-cell costimulation could be a factor. To know if the expression of T-cell costimulatory molecules was altered in tuberculosis, we analyzed the peripheral blood T-cell phenotype of 23 Mexican patients with pulmonary tuberculosis. There was severe CD4 (*P* < .001) and CD8 (*P* < .01) lymphopenia and upregulation of costimulatory molecule CD30 on CD4 and CD8 T cells (*P* < .05); this increase was higher in relapsing tuberculosis. The main finding was severe downregulation of the major costimulatory molecule CD28 on both CD8 and CD4 T cells (*P* < .001). Depletion of the CD4/CD28 subset, a hitherto undescribed finding, is relevant because CD4 T cells constitute the main arm of the cell-mediated antimycobacterial immune response.

## 1. Introduction

T cells play a central role in the response to mycobacteria. After antigen presentation by accessory cells, CD4 T lymphocytes are activated to produce IFN-*γ*, the prototypic Th1 cytokine that enhances the mycobactericidal capacity of macrophages [1]. CD8 T cells also contribute producing IFN-*γ*, lysing mycobacteria-infected macrophages, and killing bacilli through a granule-dependent mechanism [2]. Despite high immunogenicity of mycobacterial components, 17–25% of patients are anergic to tuberculin [3, 4]. Anergy can be reversed with antituberculous chemotherapy, although in some patients it may persist despite treatment [4]. Anergy might be related to multiple factors, including CD4 T-cell lymphopenia [5, 6], poor production of IL-2 and IFN-*γ* [4], defects in IFN-*γ* and IL-12 receptors [7, 8], dysregulation of the cytokine network with predominance of a Th2 response [9], and increased T cell apoptosis [10]. The causal factors involved in impaired immunity in tuberculosis (TB) are largely unknown. Some data suggest a role for ethnic and genetic factors [4, 11].

 The role of T-cell costimulation in the response to microbes has been well documented [12], although similar information about *Mycobacterium tuberculosis* (Mtb) infection is scanty. In particular, little is known about the status of the CD28/B7 pathway, which is crucial for activation of Th1 lymphocytes the main arm of the immune response against mycobacteria [1, 2]. *In vitro* studies have shown that mycobacteria upregulate the expression of costimulatory molecules by host cells [13], although the opposite has been also reported [14]. In human TB, B7-1, B7-2 can be seen in lung granuloma macrophages [15], and by flow cytometry depletion of the CD8/CD28 subset has been demonstrated [16]. The importance of the CD28/B7 costimulation pathway is highlighted by a recent study with B7DKO mice that were highly susceptible to chronic mycobacterial infection, this susceptibility being due to impaired Th1 T-cell responses [17].

 The main purpose of this study was to determine the status of T-cell costimulatory molecules in TB. For this, we carried out a flow cytometry analysis of peripheral blood T cells in a group of 23 Mexican patients with active pulmonary TB. We analyzed, together with lineage and activation markers, costimulatory molecules CD28, CD40L, CD30, CD30L, and CD27.

## 2. Study Population and Methods

### 2.1. Study Population

Twenty-three HIV-1 negative patients with active pulmonary TB were studied. Patients were admitted to the outpatient clinic of the Hospital de Infectologia, Centro Medico La Raza (IMSS) in Mexico City. The evolution time since the appearance of clinical symptoms varied from 2 to 88 months. The diagnosis, established on clinical and radiological grounds, was confirmed by acid-fast smears and culture of sputum in all cases. In all patients drug resistance tests of isolated strains were carried out. Patients were treated with isoniazid, rifampicin, and ethambutol. As controls, cells were obtained from 15 HIV negative healthy individuals of similar ages and sex distribution as the TB group. Patients and controls gave informed consent to carry out all studies, including tests for HIV.

### 2.2. Monoclonal Antibodies

 Monoclonal antibodies (mAbs), unlabeled or labeled with fluorescein isothiocyanate, phycoerythrin, or phycoerythrin-Cychrome-5 were obtained. From Serotec (Raleigh, NC, USA) mAb against CD3 (clone UCHT1, IgG1), CD45RA (clone F8-11-13, IgG1), CD30L (clone MB1, IgG2b), CD40L (clone TRAP1.3.6, IgG1), and CD27 (clone LT27, IgG2a). From Pharmingen (San Diego CA, USA) mAb against CD4 (clone RPA-T4, IgG1), CD28 (clone CD28.2, IgG1), CD95 (clone DX2, IgG1), and CD45RO (clone UCHL1, IgG2b). From Dako Corporation (Carpinteria CA, USA) we obtained mAb against CD4 (clone MT310, IgG1), CD8 (clone DK25, IgG1), and CD30 (clone Ber-H2, IgG1). Appropriate isotype control antibodies were employed.

### 2.3. Flow Cytometry Assay

 Blood samples were obtained from patients and controls by venipuncture in vacutainer tubes (Becton Dickinson, San Jose CA, USA) with EDTA as anticoagulant. All patients had been in chemotherapy for various lengths of time when the blood sample was obtained. Peripheral blood mononuclear cells (PBMCs) were isolated by centrifugation in Histopaque-1077 gradient (Sigma, St Louis MO, USA). PBMCs (5 × 10^5^) were stained with mAb or isotype control antibodies for 30 minutes, in the dark at 4°C, and washed in PBS with 1% fetal bovine serum and 0.1% sodium azide. Cells were incubated with appropriate labeled secondary antibodies for 30 min after the primary antibody. After rinsing, cells were fixed with 1% paraformaldehyde in PBS. At least 10,000 cells were analyzed in a two-color FACScan (Becton Dickinson, San Jose CA, USA) operating with CellQuest software and a 488 nm argon laser. The lymphocyte gate was set following established forward and side scatter parameters [18]. For each marker, positive values were set after eliminating with adequate controls autofluorescence and the signal elicited by the isotype control antibody.

## 3. Statistical Analysis

Data were analyzed with a Mann-Whitney nonparametric test using Graph Pad Prism version 3.02 for Windows, (Graph Pad Software, San Diego CA, USA).

## 4. Results and Discussion

In view of the lack of information about the causal factors involved in the poor T-cell response frequently found in the TB patient, we carried this flow cytometry study which aimed to know the status of T-cell costimulatory molecules. The main clinical and laboratory data of the 23 patients included in this study are shown in Table 1. Total white blood cell counts were performed on all samples using an automated cell counter and differential leukocyte counts were done by Giemsa staining. The normal ranges for WBC are WBC 4–11 × 10^3^/mm^3^; lymphocytes 16%–45%; polymorphonuclear leukocytes 45%–74%; monocytes 4%–10%.

Eleven of the 23 TB patients (47.8%) had relapsing disease; these patients had completed chemotherapy and were readmitted with recurrent respiratory symptoms due to reactivation of lung TB as revealed by chest x-ray and acid-fast staining and/or culture of sputum. In 16 patients, multidrug Mtb resistant (MDR) strains were isolated. This high number of resistant strains is due to the fact that patients were recruited from a referral infectious disease hospital. In Mexico, 5.8% of all TB cases are due to MDR strains [19]. Ten patients were women and 13 were males, ranging from 29 to 78 years in age. The average age was 55 ± 14 years and 6 patients were elderly (65 years or more). This age distribution is in keeping with data showing increased frequency of TB in the geriatric population [20]. The incidence rate of pulmonary TB in the general population in Mexico in 1994 was 14.9/100,000, and in subjects of 65 years or older it was 45.8/100,000 [21]. Since old individuals frequently present variations in T-cell phenotype [22], in order to have a similar average age as the TB group, elderly individuals were included in the control group. The average age of controls was 48 ± 19, the range from 21 to 81 years and 3 controls were elderly. In Table 2, clinical and laboratory data of control individuals are shown. No significant differences between controls and TB patients were found regarding age, sex, and white blood cell counts.

**Figure 1:**
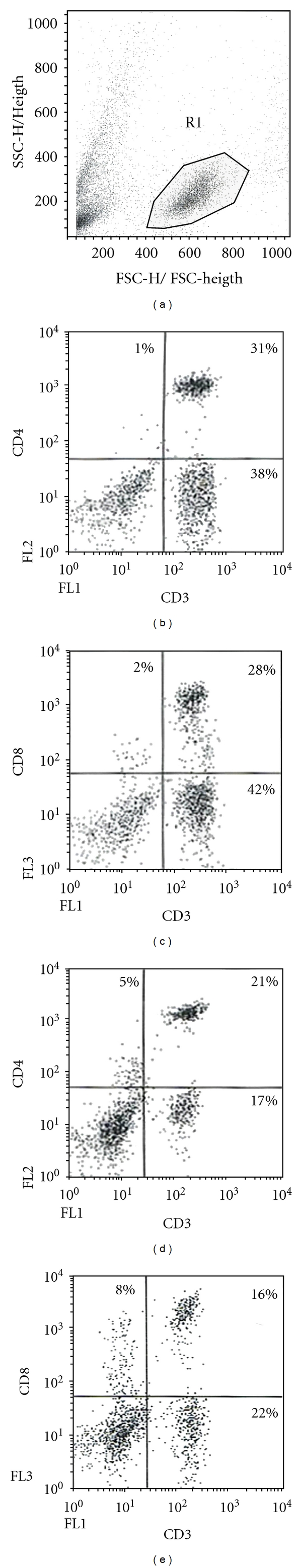
Representative forward by side scatter distribution for enriched lymphocytes used in our flow cytometry analysis is shown (a). Representative dot plots to show differences between CD3/CD4 and CD3/CD8 populations of controls (b, c) and tuberculosis patients (d, e).

In Figure 1(a), a representative forward by side scatter distribution for enriched lymphocytes used in our flow cytometry analysis is shown. In the same Figure 1 are shown representative dot plots with the percentages of CD3/CD4 and CD3/CD8 cells from controls (b, c) and TB patients (d, e). In Table 3, the numeric expression of all flow cytometry findings is presented. There was depletion of T cells with the pan T-cell marker CD3 (*P* < .05). Absolute numbers of lymphocyte subsets were calculated multiplying the percentage of a determined subset as shown by flow cytometry by the absolute lymphocyte count obtained with an automated hematology analyzer. CD4 T-cell counts were severely diminished in both absolute number and percentage (*P* < .001). CD8 T cells were also diminished but to a lesser extent (*P* < .01, absolute number and percentage). It is of note that the level of CD4 T-cell depletion in some patients was comparable to that seen in AIDS patients [23]. There was no association between age and CD4 or CD8 lymphopenia or between first-time and recurrent TB. The factors involved in T-cell depletion are poorly known. The recovery of T-cell counts in some patients after chemotherapy suggests a direct effect of mycobacteria [24]; sequestration of T cells in infected tissues has been also entertained [25]. Reduction of CD4 and CD8 T cells might influence the outcome of infection; these cells play a central role in antimycobacterial immunity, as shown by depletion with monoclonal antibodies [26], or by adoptive transfer in murine models of TB [27]. Similarly, the loss of CD4 T cells in HIV infection dramatically augments susceptibility to TB [23]. In a study, decreased CD4 T-cell counts were associated with severe disease and higher mortality [6]. In our study it was not possible to evaluate the impact of lymphopenia on the outcome of the infection, since followup of patients was insufficient. To obtain information about the activation and memory status of T cells, we analyzed CD45RA, a marker of naïve T-cells, and CD45RO that characterizes memory/effector T cells [28]. CD45RA was within normal values while CD8/CD45RO cells were diminished in absolute number (*P* < .05). As far as the CD4/CD45RO subset, there appeared to be a decreased absolute number of these cells in the TB group compared with healthy controls (controls 486 ± 42; TB 345 ± 39), although this tendency did not reach statistical significance. In contrast, the percentage of CD4/CD45RO T cells was significantly increased (*P* < .01) indicating CD4 T cell-activation, an immune response characteristic of tuberculosis infection [1]. This finding is in keeping with a flow cytometry analysis of cells recovered from tuberculous lungs by bronchoalveolar lavage showing that the great majority of CD4 T cells were CD45RO+ [29]. Contrary to that reported in the literature [10, 30], CD4 and CD8 T cells expressing the proapoptosis molecule CD95 were not significantly different in absolute numbers and percentage from those of healthy controls.

With respect to T-cell costimulatory molecules, we analyzed CD28, a member of the immunoglobulin superfamily [31], CD27, CD30, CD30L, and CD40L, which belong to the TNF family [32]. Results are shown in Table 3. No statistically significant differences were found between patients and controls regarding CD27 and CD40L. An interesting observation of this study was the increased expression of CD30 on CD4 and CD8 T cells subsets (percentage *P* < .05). CD30L was increased only in percentage in CD8 T cells (*P* < .05). These observations are in agreement with studies showing that Mtb is a potent inducer of CD30 [33]. In tuberculous pleurisy and lung granulomas, CD30 T cells are abundant [33] and high levels of soluble CD30 are found in sera of TB patients [9]. Increased soluble CD30 correlates with a Th2 pattern of cytokine production by T cells, that is permissive for the mycobacterial infection [9, 34].

**Figure 2:**
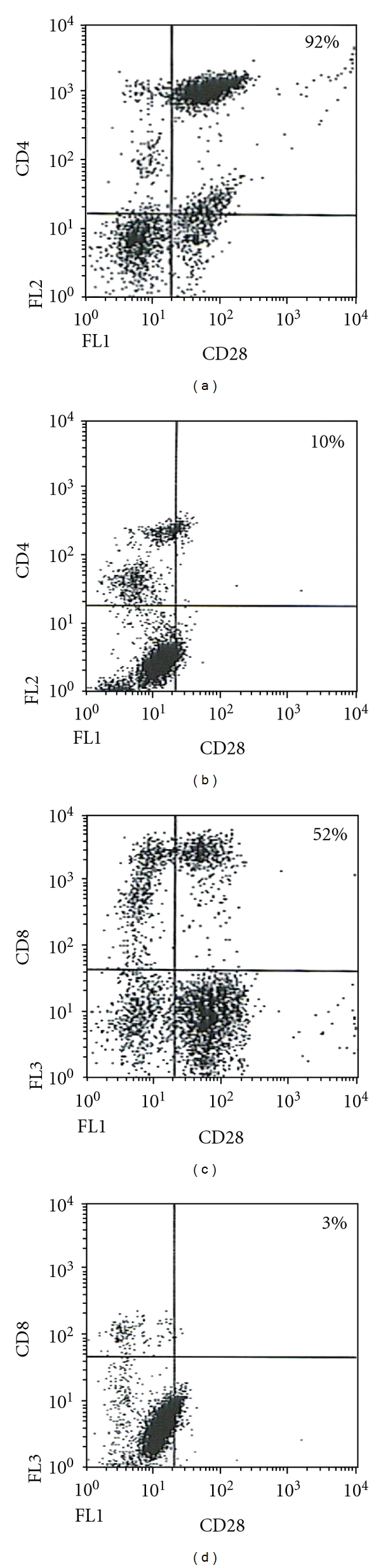
Representative dot plots to illustrate the differences of CD28+ populations between controls (a, c) and TB patients (b, d).

**Figure 3:**
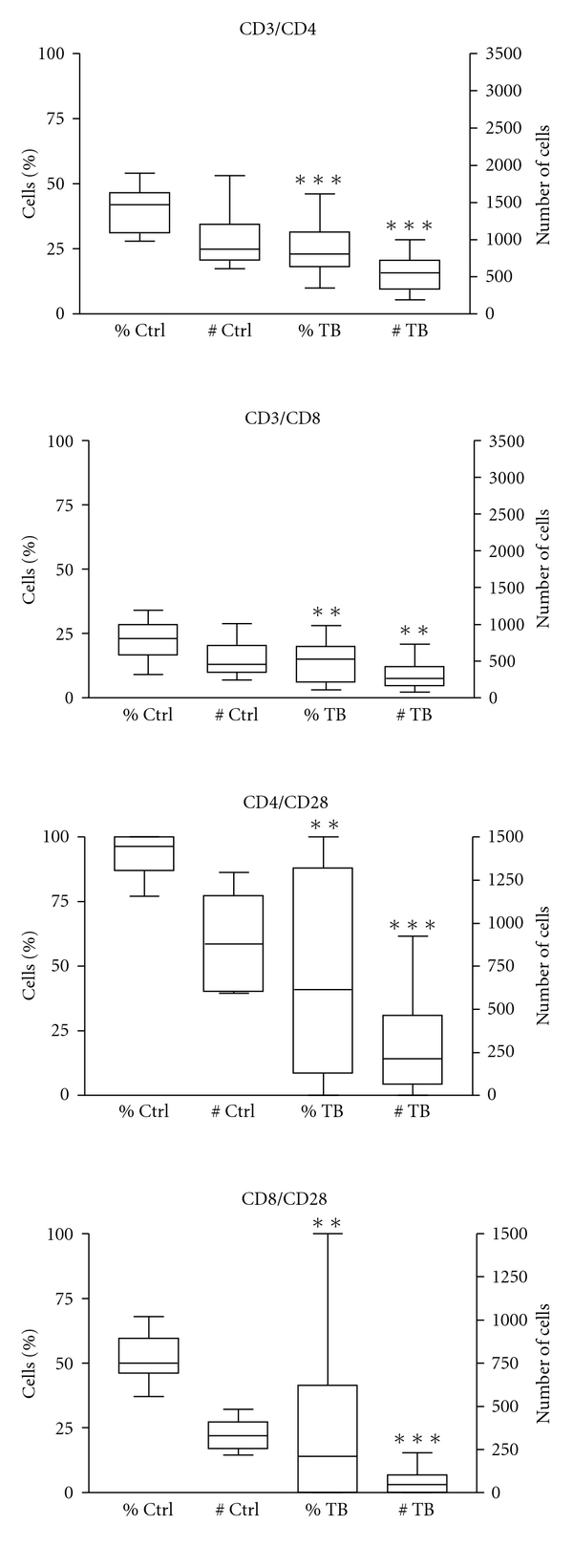
Flow cytometry analysis of peripheral blood cells from 23 patients with pulmonary tuberculosis and 15 healthy controls. Percentage and absolute numbers of CD4 and CD8 T cells are presented. The expression of CD28 on CD4 and CD8 T cells is also shown. The box plot represents the median, 25/75 percentile, and extreme values. Asterisks represent statistically significant differences. **P < .01, ***P < .001.

 Regarding CD28, the best studied costimulatory molecule, results are shown in Table 3 and Figures 2 and 3, the latter to show data dispersion. There was marked depletion of the CD8/CD28 subpopulation which was highly significant in both absolute number (*P* < .001) and percentage (*P* < .01); decreased expression of the costimulatory molecule on CD8 T cells of TB patients has been reported before [16, 30]. In a study with Ugandan patients, which were much younger than our patients [30], depletion of the CD8/CD28 subset was not associated with lymphopenia and there was increased expression of the proapoptosis molecule CD95 and decreased interferon-gama production; another difference was that CD28 expression on CD4 T cells was not diminished. These differences could be related to ethnic factors or to differences in the age of the patients included in the studies. Downregulation of CD28 on CD8 T cells is interesting in view of the important role that CD8 T cells play in antimycobacterial immunity [1, 2]. However, the significance of CD28 on the activation of CD8 T remains ill defined; a strict CD28 dependence has been documented [35] while in other studies activation of CD8 was shown to proceed normally in the absence of CD28 costimulation [36]. CD8/CD28 negative T cells differ from CD8/CD28 positive T cells in their high catalytic activity, elevated interferon-gamma production, and increased tendency to apoptosis [37].

The main finding of this study was the highly reduced counts of CD4/CD28 T cells (Table 3, Figures 2 and 3); this was highly significant in absolute number (*P* < .001) and percentage (*P* < .01). Downregulation of CD28 on CD4 T cells of TB patients has not been, to our knowledge, previously reported. This finding is potentially important since the interaction of CD28 with its ligand B7 is crucial to activate T cells [31, 38]. T-cell activation in the absence of CD28 results in anergy [38], a phenomenon present in 17–25% of TB patients [3, 4]. There are few studies on the status of the CD28/B7 costimulation pathway in TB. A study showed that dendritic cells challenged with Mtb upregulate costimulatory molecules, including B7.1 [13], although, in mycobacteria-infected macrophages, decreased expression of B7.1 and B7.2 has been documented [14]. In human TB, B7-1, B7-2, and CD40 are demonstrated in macrophages infiltrating granulomas [15]. Recently, it was shown that B7DKO mice were very susceptible to mycobacterial infection due to defective Th1 immunity [17]. Studies with other pathogens support the importance of CD28 in antimicrobial immunity. CD28 (−/−) mice are highly susceptible to *Chlamydia trachomatis* and *Salmonella typhimurium* infection [39, 40].

 Regarding clinical data, a history of diabetes, and hypertension had no impact on the T cell phenotype of our TB patients. No differences were found between age and CD28 downregulation. We could not obtain enough data to asses a relationship between extent of disease and CD28 depletion. However, differences were found when comparing first-time TB with relapsing infection, a variety of TB characterized by lack of compliance to chemotherapy, frequent isolation of MDR strains, and a poor outcome [41, 42]. First, 10 of the 11 patients (90.9%) with relapsing TB had MDR strains while this was found in 6 of 12 first-time TB patients (50%). A significant difference was found in WBC counts (relapsing TB, 5675/mm^3^; first-time TB, 7053/mm^3^; *P* < .05). In addition, in relapsing infection CD4 lymphopenia was less pronounced (*P* < .05), the percentages and absolute numbers of CD4/CD27 cells were increased (*P* < .05), and the number of CD4/CD30 cells was also increased (*P* < .05). No apparent differences were found regarding the expression of CD28 between relapsing and first-time TB.

## 5. Conclusions

In summary, in this descriptive flow cytometry study, we have observed severe phenotypic abnormalities of T cells in patients with active pulmonary tuberculosis including severe depletion of CD4 and CD8 T-cell counts. Importantly, we observed markedly decreased expression of the major T-cell costimulatory molecule CD28 on both CD8 and CD4 T cells which together with the marked CD4 lymphopenia could be causal factors in the inadequate cell-mediated immune responses that frequently complicate the course of TB. Further studies are warranted to determine if treatment influences the T-cell phenotypic changes described in this paper.

## Figures and Tables

**Table 1:** Clinical and laboratory data in patients with tuberculosis.

Patient	Age (years)	Sex	Relapses	Duration (months)	Strain	WBC (mm^3^)	Lymph (%)	Mono (%)	PMN (%)
01	39	F	—	12	R	5500	20	3	77
02	49	F	—	12	S	4800	30	3	67
03	49	M	√	24	R	6900	28	1	71
04	44	M	√	22	R	13700	23	2	75
05	47	M	—	10	S	5700	24	2	74
06	64	M	√	63	R	6800	32	3	65
07	57	M	√	16	S	4200	32	1	67
08	75	M	—	12	R	6700	22	1	77
09	78	M	—	22	R	6500	24	5	71
10	30	F	√	36	R	7200	28	2	70
11	61	F	√	55	R	8000	29	5	66
12	29	F	√	12	R	10700	17	1	82
13	58	M	√	12	R	5700	42	1	57
14	58	F	—	6	S	6400	30	1	69
15	68	F	—	4	S	4100	44	0	56
16	63	M	√	36	R	6700	32	8	60
17	40	F	—	18	R	5700	34	17	49
18	71	M	√	88	R	8500	25	6	69
19	54	F	—	60	R	5600	45	10	45
20	47	M	—	2	S	3600	34	5	61
21	74	M	√	36	R	7100	33	16	51
22	66	F	—	9	ND	7200	28	2	70
23	46	M	—	12	R	6300	55	7	38

*M. tuberculosis* strains, resistant (R), susceptible (S), ND, not done. WBC, white blood cells; Lymph, lymphocytes; Mono, monocytes; PMN, polymorphonuclear leukocytes. Duration refers to the duration of the tuberculosis pulmonary symptoms as stated in the clinical records.

**Table 2:** Clinical and laboratory data in control individuals.

	Age (years)	Sex	WBC (mm^3^)	Lymph (%)	Mono (%)	PMN (%)
01	39	F	4750	39	1	60
02	33	M	ND	ND	ND	ND
03	34	F	4670	34	7	53
04	21	F	5800	48	2	50
05	64	M	5050	41	7	52
06	61	M	7760	32	7	61
07	74	M	9900	22	10	68
08	81	M	5880	24	8	68
09	31	M	9750	20	0	80
10	63	F	6550	28	5	67
11	22	F	8000	39	0	61
12	23	M	5900	38	1	60
13	56	F	5990	28	5	67
14	51	M	7890	23	6	71
15	68	M	6670	24	9	67

F, female; M, male; ND, not done; WBC, white blood cells; Lymph, lymphocytes; Mono, monocytes; PMN, polymorphonuclear leukocytes.

**Table 3:** Phenotype of peripheral blood T lymphocytes in 23 patients with active pulmonary tuberculosis and in 15 healthy controls.

Marker	Controls		TB Patients	
	%	#	%	#
CD3	66 ± 4	1600 ± 137	56 ± 5*	1263 ± 161*
CD3/CD4	39 ± 82	948 ± 90	24 ± 2***	521 ± 47***
CD3/CD8	22 ± 2	520 ± 56	14 ± 2**	286 ± 36**
CD4/CD40L	4 ± 1	32 ± 10	11 ± 3	30 ± 7
CD8/CD40L	4 ± 3	21 ± 15	12 ± 6	56 ± 25
CD4/CD45RA	17 ± 4	212 ± 37	32 ± 5	206 ± 43
CD8/CD45RA	61 ± 7	330 ± 48	58 ± 7	265 ± 59
CD4/CD45RO	52 ± 5	486 ± 42	73 ± 4**	345 ± 39
CD8/CD45RO	47 ± 5	244 ± 39	45 ± 6	153 ± 29*
CD4/CD30	1 ± 0.5	8 ± 4	6 ± 2*	28 ± 12
CD8/CD30	1 ± 0.3	1 ± 1	5 ± 2*	15 ± 7
CD4/CD30L	6 ± 1	60 ± 12	23 ± 8	98 ± 23
CD8/CD30L	3 ± 1	17 ± 10	13 ± 3*	60 ± 21
CD4/CD28	93 ± 2	885 ± 82	48 ± 8**	268 ± 54***
CD8/CD28	51 ± 3	325 ± 26	24 ± 6**	63 ± 14***
CD4/CD95	43 ± 8	425 ± 86	54 ± 7	298 ± 49
CD8/CD95	49 ± 7	280 ± 51	40 ± 8	155 ± 38
CD4/CD27	42 ± 10	401 ± 107	50 ± 9	275 ± 56
CD8/CD27	32 ± 8	154 ± 40	29 ± 7	81 ± 20

Media absolute cell counts and percentage ± SD of flow cytometry, results. Statistically significant differences between controls and tuberculosis patients were determined by a Mann-Whitney nonparametric test. *P < .05, **P < .01, ***P < .001.
